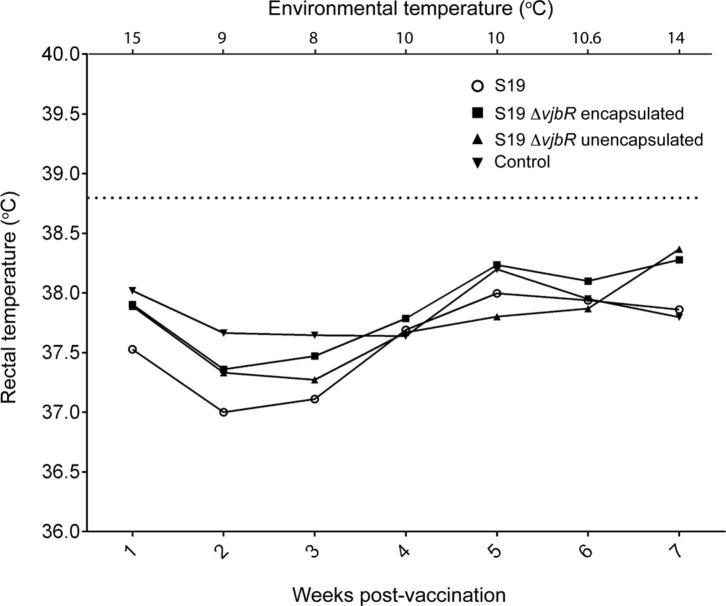# Vaccine safety studies of *Brucella abortus* S19 and S19Δ*vjbR* in pregnant swine

**DOI:** 10.1016/j.jvacx.2019.100041

**Published:** 2019-08-22

**Authors:** Slim Zriba, Daniel G. Garcia-Gonzalez, Omar H. Khalaf, Lance Wheeler, Sankar P. Chaki, Allison Rice-Ficht, Thomas A. Ficht, Angela M. Arenas-Gamboa

**Affiliations:** a Department of Veterinary Pathobiology, College of Veterinary Medicine & Biomedical, Sciences, Texas A&M University, College Station, TX, USA; b Department of Veterinary Pathology & Poultry Diseases, College of Veterinary Medicine, University of Baghdad, Baghdad, Iraq; c Department of Molecular and Cellular Medicine, Texas A&M Health Science Center, College Station, TX, USA

**Keywords:** *Brucella*, Swine, Vaccine safety, Brucellosis, *B. abortus* S19, *B. abortus* S19Δ*vjbR*

## Abstract

Brucellosis in swine is caused by *Brucella suis,* a bacterial infection of nearly worldwide distribution. *Brucella suis* is also transmissible to humans, dogs and cattle and is considered a reemerging disease of public health concern. To date, there is no effective vaccine for swine. This prompted us to investigate the potential use of the commercially available vaccine for cattle or the live attenuated vaccine candidate S19Δ*vjbR*. As the first step, we sought to study the safety of the vaccine candidates when administered in pregnant sows, since one of the major drawbacks associated with vaccination using Live Attenuated Vaccines (LAV) is the induction of abortions when administered in pregnant animals. Fifteen pregnant gilts at mid-gestation were divided into four groups and subsequently vaccinated subcutaneously using different formulations containing 2.0 ± 0.508 × 10^9^ CFU of either S19 or S19Δ*vjbR*. Vaccination in pregnant animals with the vaccine candidates did not induce abortion, stillbirths or a reduction in litter size. Multiple tissues in the gilts and piglets were examined at the time of delivery to assess bacterial colonization and histopathological changes. There was no evidence of vaccine persistence in the gilts or bacterial colonization in the fetuses. Altogether, these data suggest that both vaccine candidates are safe for use in pregnant swine. Analysis of the humoral responses, specifically anti-*Brucella* IgG levels measured in serum, demonstrated a robust response induced by either vaccine, but of shorter duration (4–6 weeks post-inoculation) compared to that observed in cattle or experimentally infected mice. Such a transient humoral response may prove to be beneficial in cases where the vaccine is used in eradication campaigns and in the differentiation of vaccinated from infected animals. This study provides evidence to support future efficacy studies of both vaccine candidates in swine.

## Highlights

• Vaccination with *Brucella abortus* S19 or S19Δ*vjbR* in pregnant swine did not induce abortion, stillbirths or a reduction in litter size.
• Gross and histopathological evaluation did not demonstrate any local or systemic side effect associated with either vaccine.
• At the time of the delivery, there was no evidence of the presence of either vaccine strains in the fetuses, placentas or sows.
• Both vaccine candidates are safe for use in pregnant swine.

## 1 Introduction

Brucellosis is a zoonosis caused by *Brucella* spp. of nearly worldwide distribution. Among the 12 identified species classified based on preferential host specificity, 3 are highly pathogenic for their preferred host species [*Brucella melitensis* (sheep and goat), *Brucella abortus* (cattle) and *Brucella suis* (swine)], as well as humans, and are all associated with significant economic losses in different parts of the world [1]. Brucellosis in humans is acquired by direct contact with infected animal tissues or consumption of unpasteurized milk products. It is considered a debilitating disease with undulant fever as a major symptom, frequently accompanied by fatigue, sweats, malaise, weight loss and arthralgia [2]. Several complications can be encountered from chronic infection including osteoarticular, cardiovascular, neurological and adverse obstetrical outcomes [3]. *Brucella* infection in animals is characterized by abortion and infertility in domestic and wildlife animals [4]. Infection usually leads to major economic losses, with a significant negative impact [1].

The United States (US) is free from the disease in domestic swine in contrast to feral pigs in which the numbers of infected animals are on the rise [5]. Considering the expanding number of feral swine and the potential for spillover to livestock, the development of improved countermeasures to prevent the reemergence of the disease in domestic animals is of paramount importance [6]. Swine brucellosis is the perfect example of a “One Health” approach, in which vaccination represents a key strategy to protect animals and ultimately humans [7]. In the absence of a protective vaccine, this study evaluates the potential use of S19 and S19Δ*vjbR* vaccine candidates in swine. We have previously tested S19Δ*vjbR* vaccine strain in multiple animal species and have demonstrated an added effect in terms of reduction of inflammatory response or tissue colonization, therefore reducing the possible side effects associated with vaccination of S19 in pregnant animals. Specifically, we describe the safety profile of both vaccine candidates using different delivery systems when inoculated into pregnant sows, since abortion secondary to vaccination is a common and undesired side effect observed when using Live Attenuated Vaccine candidates (LAV) for brucellosis. We also sought to investigate the potential of vertical transmission by performing bacteriological and histopathological analysis of maternal and fetal tissues. Finally, humoral responses induced by the vaccine formulations were characterized as the first step towards understanding immune protection induced by LAVs against brucellosis in swine.

## 2 Materials and methods

### 2.1 Animals

American Yorkshire healthy gilts were used for this study and confirmed to be negative for brucellosis by ELISA. Gilts were synchronized and artificially inseminated to generate pregnancies that were at the same gestational age during vaccination. Only pregnant animals, confirmed via ultrasound, were included in the study. All animal procedures were performed under TAMU Institutional Animal Care and Use Committee (IACUC) guidelines.

### 2.2 Vaccine strains

The S19 Δ*vjbR* vaccine strain was engineered and used as a vaccine candidate in a previous study [8]. The *Brucella abortus* S19 was obtained from the National Veterinary Services Laboratories (NVSL, Ames, IA). Both strains were grown on tryptic soy agar plates (TSA) for 3 days at 37 °C with 5% (v/v) CO_2_ and harvested from the surface of the plates using phosphate-buffered saline (PBS), pH 7.2. A dose of 2.0 ± 0.508 × 10^9^ CFU/ animal was used as confirmed by actual viable colony counts of bacterial serial dilutions on TSA plate.

### 2.3 Encapsulation of *B. abortus* S19 Δ*vjbR* vaccine

Alginate encapsulation was performed as previously described [8]. *B*. *abortus* S19Δ*vjbR* was resuspended in 1 ml of MOPS buffer (10 mM MOPS, 0.85% NaCl [pH 7.4]) and mixed with 5 ml of alginate solution in MOPS buffer of pH 7.3. Extrusion of the suspension through a 200-μm nozzle into a 100 mM calcium chloride solution produced capsules under continuous stirring. Capsules were crosslinked in poly-l-lysine and coated with 2.5 mg of VpB (vitelline protein B), followed by the addition of an outer layer of alginate [8].

### 2.4 Immunization of pregnant gilts

Pregnant gilts were randomly distributed into 4 groups and inoculated at mid -gestation (60–66 days of gestation) subcutaneously (SQ) in the scapular area with a single dose containing 2.0 ± 0.508 × 10^9^ CFU of either (1) S19 (n = 4), (2) encapsulated S19Δ*vjbR* (n = 4), (3) unencapsulated S19Δ*vjbR* (n = 4), or (4) empty capsules/control (n = 3).

### 2.5 Clinical evaluation of gilts

Prior to vaccination and until delivery, gilts were monitored twice a day for side effects associated with vaccination including abortion, adverse reactions at the injection site, food consumption, vaginal discharges, and fever. Rectal temperature of pregnant gilts was measured daily. A temperature of 38.8 °C (±0.3 °C) was considered the normal body temperature threshold for gilts [9].

### 2.6 Vaginal shedding of the vaccine strains

Screening for vaginal shedding was performed biweekly on all animals. Vaginal swabs were plated onto Farrell’s agar medium for bacterial isolation. Plates were incubated at 37 °C and monitored daily for up to 30 days.

### 2.7 Postmortem examination of gilts

Within the first 3 days post-delivery, all sows were euthanized via pentobarbital overdose and necropsied for the detection of any gross changes associated with vaccination. Tissue sections including spleen, liver, lung, uterus, placenta, pre-scapular, mammary, inguinal and mesenteric lymph nodes were collected and formalin fixed. Paraffin sections were H&E stained and examined by a board-certified veterinary anatomic pathologist.

### 2.8 Bacterial colonization in tissues from gilts

Colonization of maternal tissues by the different vaccine formulations was assessed by culture after delivery. Spleen, liver, lung, uterus, pre-scapular, mammary, inguinal and mesenteric lymph nodes were removed and weighed. One gram of tissue from each organ was homogenized in 1 ml of phosphate buffered saline, pH 7.2 (PBS) and cultured on TSA/ and Farrell’s agar media and incubated up to 30 days at 37 °C with 5% (v/v) CO_2_. Bacterial colony forming unit (CFU) was measured by visual enumeration.

### 2.9 Postmortem examination of piglets

Piglets were euthanized and a full necropsy was performed within the first hours of birth. Lung float test was conducted to assess fetal viability upon birth. Tissue sections of spleen, liver, lung and umbilical cord were fixed in 10% (v/v) buffered formalin, paraffin embedded and sections were stained with hematoxylin and eosin (H&E). Histological changes were assessed by a board-certified veterinary anatomic pathologist.

### 2.10 Bacterial colonization of piglet tissues

Within the first 12 h of delivery, sections of spleen, lung, liver, kidney, stomach, and umbilicus were collected from all piglets. Homogenized tissue was cultured on TSA and Farrell’s agar media and incubated up to 30 days at 37 °C with 5% (v/v) CO_2_. A total of 181 piglets were examined and all cultures were done in duplicate.

### 2.11 Humoral responses in pregnant gilts. Rose Bengal Test (RBT)

Serum agglutination against *Brucella* antigen was performed (USDA/APHIS, NVSL). 30 μl of sera from immunized animals were added to an equal volume of Rose Bengal antigen onto a card and mixed thoroughly. A scale was developed to categorize the degree of agglutination as; (1) ++++/+++ strong, (2) ++ mild, (3) + weak and (4) − no agglutination.

### 2.12 Determination of anti-*Brucella* IgM and IgG antibodies

iELISA was performed at 0, 2, 4- and 6-weeks post-vaccination. 96 well plates were coated with heat killed and sonicated *Brucella abortus* lysate (250 ng/well) overnight at 4 °C. Following blocking (0.25% [w/v] bovine serum albumin in 10 mM PBS containing 0.05% (v/v) tween 20) for 2 h at room temperature, plates were washed and incubated with sow sera samples (diluted 1:500 in the blocking buffer) for 1 h at 37 °C. Following washing, peroxidase-conjugated secondary antibody (goat anti-swine IgG or IgM, KPL) was added at a dilution of 1:1000 in blocking buffer and incubated at 37 °C for 1 h. Following washing, horseradish peroxidase substrate was added for 15 min and OD was measured at 450 nm. All assays were performed in triplicate.

### 2.13 Statistical analysis

All analyses were performed using the GraphPad Prism 6.0 software (San Diego, CA, USA) and *P* values <0.05 were considered significant. The significance of differences between the different clinical parameters including litter size, abortions, CFU and fever were analyzed by ANOVA followed by Tukey post-hoc analysis. The two-way analysis of variance (ANOVA) test was used for the anti-*Brucella* IgG and IgM experiments followed by Tukey’s multiple comparisons test.

## 3 Results

### 3.1 Clinical findings and pregnancy outcomes

One of the main drawbacks associated with vaccination using LAV is the induction of abortion in pregnant animals [10], [11]. In order to determine if vaccination with either S19 or S19Δ*vjb*R induced abortions when given to pregnant animals, sows were vaccinated subcutaneously (SQ) at a dose of 2.0 ± 0.508 × 10^9^ CFU with the different vaccine candidates at mid-gestation and were monitored daily until delivery. No adverse side effects were observed during the course of the study. The duration of pregnancy was 112 to 116 days with no significant differences between groups (Table S1). There was no significant difference in litter size, abortions, mummies or stillbirths among the different groups (Table 1). The mean litter size for S19 was 12.25 piglets/ sow, for S19Δ*vjbR* unencapsulated was 11.25 piglets/sow, for S19Δ*vjb*R encapsulated was 12.5 piglets/sow and for the control was 12.7 piglets/sow.

**Table 1:** Litter size from pregnant gilts and description of the number of abortions, postpartum deaths and stillbirths.

Group	Animal Number	# of piglets per gilt	Total litter size	Litter size (Mean ± SD)^a^	# of abortions^b^	# of post- partum deaths^c^	# of Stillbirth^d^	# of mummified^e^
S19	1	13	49	12.25 ± 2.5	0/49	3		
	2	12				1	1	
	3	9				2		1
	4	15				0		

S19 Δ*vjbR* encapsulated	1	13	49	12.25 ± 0.96	0/49	1		
	2	12				2		
	3	11				0		
	4	13				0		

S19 Δ*vjbR* unencapsultated	1	12	45	11.25 ± 2.99	0/45	1		
	2	8				1		
	3	15				1		
	4	10				2		

Control	1	11	38	12.7 ± 3.79	0/38	10		
	2	10				0		
	3	17				2	1	1

a *p-value* of 0.9.

b Abortion is defined by the expulsion of dead fetuses prior to normal delivery (normal delivery is estimated to occur at 115 days of pregnancy in swine).

c Postpartum deaths are defined as delivery of normal piglets with subsequent death secondary to trauma by crushing or filial infanticide.

d Stillbirths were classified as piglets who were delivered without signs of life with confirmed death during pregnancy.

e Mummified are classified as fetuses delivered with signs of decomposition (autolyzed).

Gilts were also monitored daily to evaluate clinical signs associated with vaccination. Throughout the study period, no changes in behavior, loss of body weight, or local inflammation response at the injection site were observed among the different groups (*data not shown*). No significant changes in body temperature were observed between groups regardless of the vaccine formulation or vaccine strain (Fig. S1). Although not significant, decreases in body temperature in all groups were observed at 2- and 3-weeks post-vaccination, which corresponded with low environmental temperatures at the time of the study.

### 3.2 Vaccine shedding in vaginal secretions

A major drawback of the use of LAV is the possibility of vaccine excretion into the environment, serving as a potential source of contamination and infection of naïve animals or non-target species [12]. In an attempt to determine if any of the vaccine formulations were shed into the environment through vaginal secretions, vaginal swabs cultures were performed biweekly starting from the day of vaccination until delivery. Although, all sows were seropositive, no vaccine shedding was observed in any pregnant gilts through vaginal secretions.

### 3.3 Vaccine colonization in gilts

Bacterial colonization, at the time of delivery, was evaluated in multiple tissues. No bacteria were recovered from any of the tissues examined (blood, liver, lung, spleen, mammary lymph node, inguinal lymph node, uterus, and placenta) at the time of delivery regardless of the formulation and vaccine strain used.

### 3.4 Postmortem examination of gilts

**Fig. 1:**
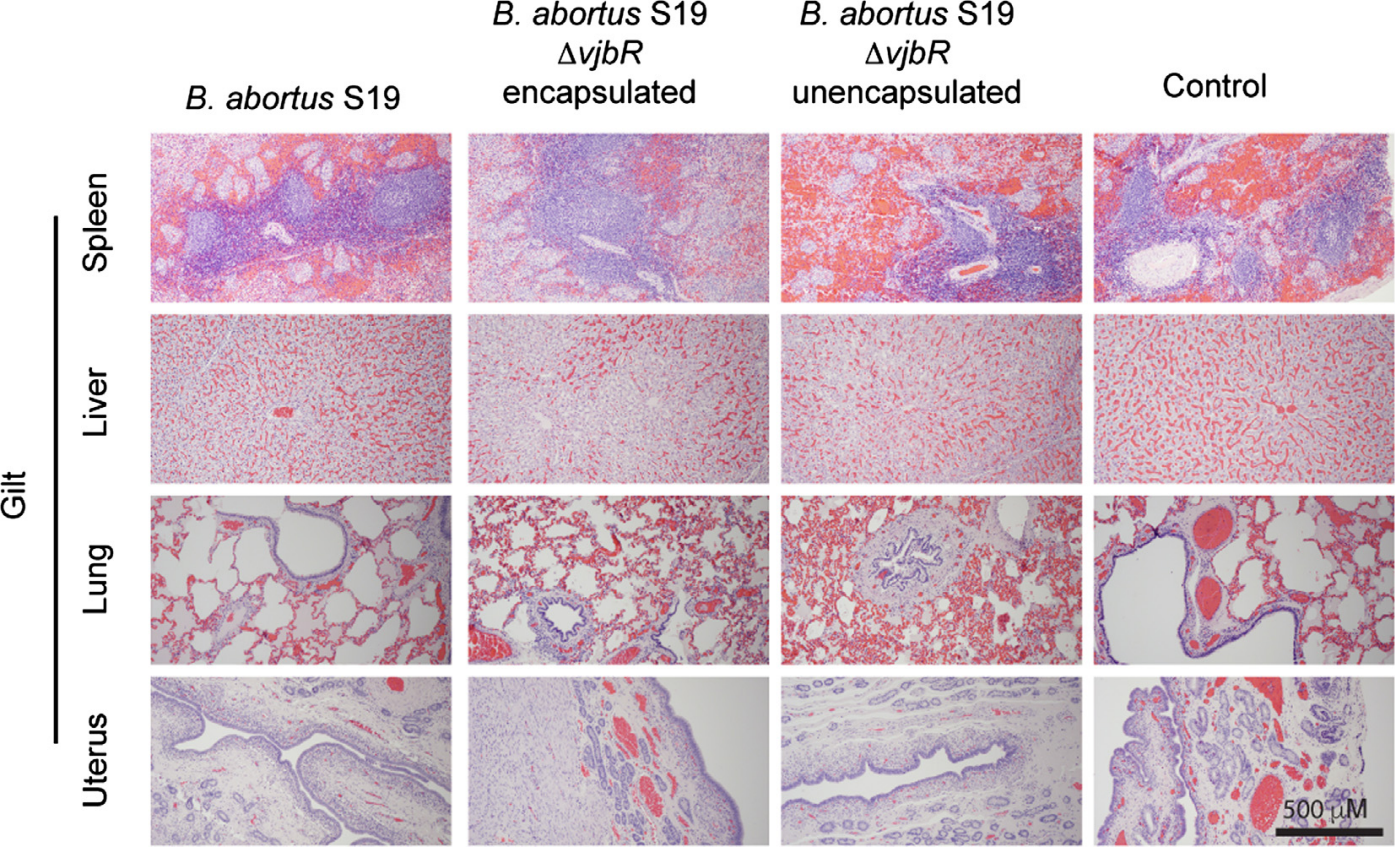
Histological analysis of spleen, liver, lung and uterus from gilts inoculated with (a) S19, (b) S19 Δ*vjbr* encapsulated, (c) S19 Δ*vjbr* unencapsulated and d) empty capsules (control group) at 5 days post-delivery. No microscopic changes were observed in any of the vaccinated animals.

Assessment of any gross and histopathological changes associated with vaccination was investigated. Following delivery, all sows were euthanized and a full necropsy was performed. No gross changes associated with vaccination in any major organ, site of inoculation or reproductive tissues was evident. Tissue sections consisting of liver, lung, spleen, uterus, and placenta were histologically normal and resembled the control group (Fig. 1).

### 3.5 Serological responses in pregnant gilts

#### 3.5.1 Rose Bengal Test

The humoral response elicited by the vaccination of gilts was evaluated biweekly over the course of 6 weeks. At 2 weeks post-vaccination, the agglutination response was highest among all groups regardless of the formulation or the vaccine strain (Table S2). There was no statistical difference between the different vaccine candidates. Starting at 4 weeks post-vaccination, the agglutination response started to decrease in all groups and varied from mild to weak agglutination, with only 2 animals from the unencapsulated S19 group still remaining as strong positives. At 6 weeks post-vaccination, regardless of the treatment, all gilts became serologically negative. One gilt vaccinated with unencapsulated S19 Δ*vjbR* remained negative to RBT during the whole period of the experiment but was later confirmed by ELISA to have seroconverted. Serum samples from control animals remained negative throughout the study.

#### 3.5.2 Evaluation of *Brucella-*specific antibody in gilts

**Fig. 2:**
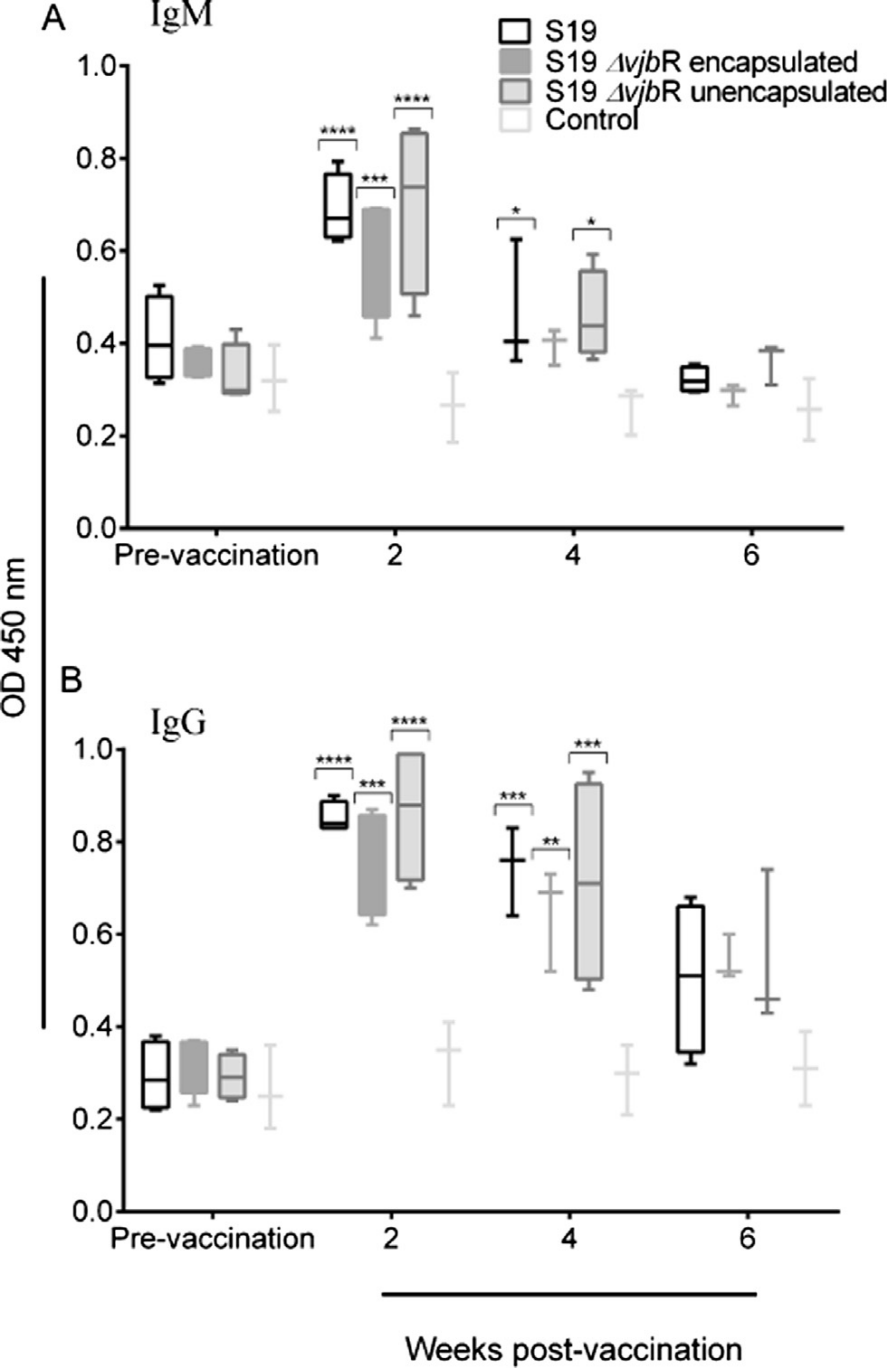
Anti-Brucella specific IgM and IgG responses in serum samples from individual gilts immunized with different vaccines (S19, S19 Δ*vjbr* encapsulated and S19 Δ*vjbr* unencapsulated) and empty capsules (control group). Results are expressed as the mean of OD values (450 nm). Statistical analysis was performed by comparing the mean of the groups using the two-way analysis of variance (ANOVA) with Tukey’s multiple comparisons test. Significant differences between vaccine treatment groups and the control group were found at week 2 and 4 post vaccination *P < 0.05, **p < 0.01, ***P < 0.001, ****P < 0.0001.

The serological findings for the vaccinated gilts are shown in Fig. 2. Serum collected at 0, 2, 4- and 6-weeks post-vaccination was assayed for the presence of *Brucella*-specific IgM and IgG antibodies by ELISA. Immunization with the S19 or S19Δ*vjbR* vaccine candidates elicited an anti-*Brucella* specific IgM and IgG response that was clearly detectable at 2 weeks post vaccination for either encapsulated or unencapsulated vaccine. Anti-*Brucella* IgM and IgG levels were similar at 2 weeks post-vaccination, however, by 4 weeks, the IgM levels were significantly reduced compared to IgG levels which persisted for 4 weeks and then started to decrease (Fig. 2A and 2B).

The variation of the results obtained between the RBT and IgG iELISA at different time points prompted us to evaluate the correlation between both approaches. The kappa value and the likelihood estimates were calculated using a Bayesian model [13]. Seroconversion of the vaccinated gilts at 2-week post-vaccination was at its highest by agreement of the iELISA and RBT (k = 0.8). The agreement between RBT and IgG iELISA tests was found substantial at 2 weeks (k = 0.8) and 4 weeks (k = 0.65) post-vaccination suggesting that RBT can be used during this interval as a valuable screening tool to monitor early vaccination (Fig. S2).

### 3.6 Fetal colonization

One of the main disadvantages of the use of LAV is the possibility of the vaccine strains to cross the placenta and colonize the fetus resulting in reproductive failure and dissemination of the vaccine strains into the environment following delivery or abortion [14]. Multiple tissues (181 fetuses) including the liver, spleen, lung, umbilicus, kidney and gastric contents were cultured. There was no evidence of fetal colonization at the time of delivery from any of the fetuses regardless of the strain or delivery system.

### 3.7 Postmortem examination of piglets

**Fig. 3:**
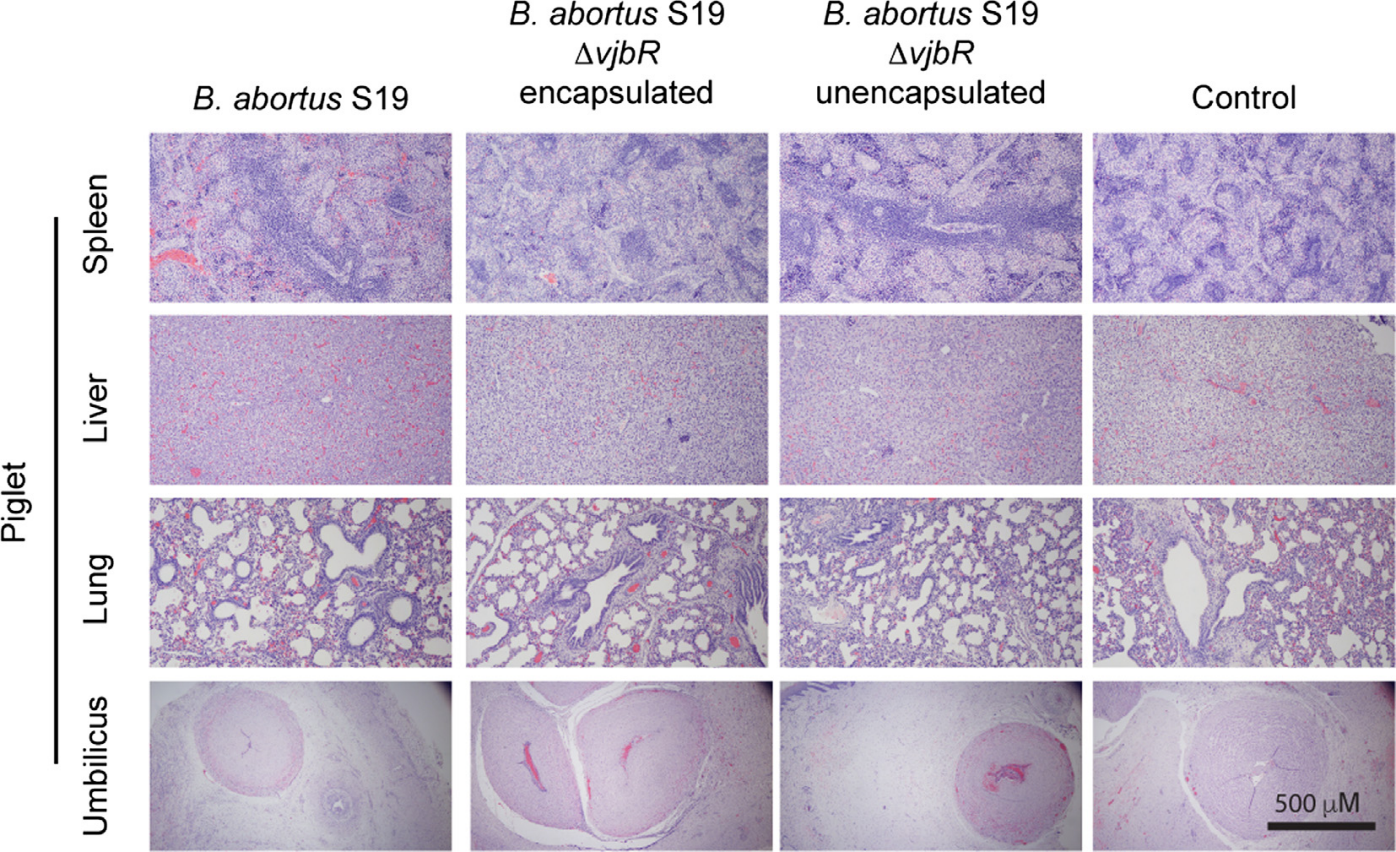
Histological analysis of spleen, liver, lung and umbilical cords from piglets of gilts inoculated with (a) S19, (b) S19 Δ*vjbr* encapsulated, (c) S19 Δ*vjbr* unencapsulated and (d) empty capsules (control group). None of the piglets inoculated with the different vaccine strains had any histopathological changes in the major organs.

Complete gross and histopathological evaluations were conducted in all piglets (181). Tissue sections from all organs were unremarkable with no differences observed between the treatment groups and controls (Fig. 3). Fetal deaths observed following farrowing and suspected to be associated with traumatic events were confirmed on gross and histopathological examination. In such cases, the presence of acute hemorrhage was consistently observed (*data not shown*). In the cases of stillbirths (total of 3), there was no evidence of an inflammatory or an infectious process and these were considered to be unrelated to the vaccination. Two of the stillborn observed were from the control.

## 4 Discussion

Despite the endemic nature of *B. suis* in the feral swine population and the imminent zoonotic threat, only a handful of studies have been performed throughout the years to develop and evaluate potential vaccine candidates for swine [15], [16], [17], [18], [19], [20]. Historically, vaccination has been recognized as the most straightforward and powerful way to control infectious diseases. The most effective programs for brucellosis in livestock (cattle, sheep, and goats) have been achieved through the use of live attenuated vaccines such as Strain 19 and RB51 in cattle, and Rev.1 in small ruminants [21], [22]. At this time, no safe and effective vaccine for swine is available. The only commercially available vaccine is the S2 vaccine developed in China [19]. Due to the significant efficacy and safety concerns including human infection and the described lack of reproducibility, the broad implementation of S2 in other countries is highly unlikely. In the absence of a vaccine against *B. suis* infection, obvious alternatives include the potential use of the commercially available vaccines for cattle (S19 and RB51) in swine. Vaccination of pregnant sows with S19 has only been described in one study conducted in a brucellosis endemic farm in 1948, in which abortions were reported to occur in pregnant swine that were vaccinated with S19 [16]. However, it was unclear from this study whether abortion was due to vaccination or a consequence of natural infection due to the endemic nature of the disease at the premises where the study was conducted. More recently, parenteral vaccination with RB51 failed to induce a humoral or cell-mediated immune response and vaccination did not protect gilts or their fetuses against *B. suis* challenge [18]. Thus, there is a critical need to develop a *Brucella* vaccine for swine that provides protection, not only against abortion but most importantly that it will prevent the organism from shedding into the environment. Ideally, this vaccine should be safe regardless of the reproductive status of the animal and should provide differentiation of vaccinated from naturally exposed animals.

Over the past years, our laboratories have been interested in the development of improved vaccines, with a strong emphasis on designing new LAV. Among these, we have developed and tested the LAV candidate designated S19Δ*vjbR* in which the *vjbR* gene (BABII 0118) is absent [8]. Safety and efficacy studies using *vjbR* mutants have revealed a significantly attenuated phenotype (reduced organism persistence, reduced inflammatory response and lack of induction of any adverse side effects in laboratory and domestic animals) while conferring high levels of protective immunity [8]. In order to enhance the efficacy of the Δ*vjbR* vaccine, part of our strategy involves the use of a delivery system designated alginate microencapsulation [8]. This approach has proven to be an effective way of enhancing the safety and efficacy of live attenuated vaccines against brucellosis [23].

As the first step towards the development of a vaccine for swine, we sought to concentrate our efforts in evaluating the safety profile by (1) assessing the potential risk of inducing abortion when inoculated into pregnant animals, (2) determining the presence of any adverse clinical symptoms associated with vaccination, (3) determining organism excretion into the environment via different routes including placenta, vaginal secretions as well as vertical transmission to the fetuses and (4) determining if there is a persistent infection in sows following delivery.

It is well-known that infection with *Brucella* spp induces abortions in livestock including swine. Under experimental conditions, *B. suis* can induce fetal death when animals are infected after day 40–45 of pregnancy with abortion occurring usually at mid to late gestation [24]. Based on these results, we wanted to vaccinate sows during the most susceptible stage of gestation and monitor for abortion. Pregnant animals were inoculated at mid-gestation and monitored daily until delivery. As expected, vaccination with either the encapsulated or non-encapsulated S19Δ*vjbR* did not induce abortion and the vaccine was incapable of colonizing either placental or fetal tissues which further supports its attenuated phenotype. It is well known that S19 vaccination induces abortions in multiple animal species. Interestingly, abortion is not significantly reduced when a lower dose of 3.0 × 10^8^ organisms is used, clearly indicating the residual virulence of S19 in cattle [25]. Additionally, a similar effect was observed after subcutaneous immunization of pregnant reindeer with 1.2 × 10^8^ CFU of *Brucella abortus* S19 [26]. Interestingly, vaccination with S19 did not induce abortion in this study. Decreased susceptibility to abortion secondary to S19 vaccination in gilts might be explained by a decreased capacity of species other than *B. suis* to establish a marked or persistent infection in pigs. Studies conducted by Stuart et al [17] demonstrated that inoculation with *Brucella abortus* wild-type Strain 544 can only establish a short-lived infection for up to 99 days post-inoculation. This is different than what is usually observed in cattle where vaccination of adult pregnant cattle with S19 induces abortions at an approximate rate of 22% when animals are inoculated with the standard dose of 5.8 × 10^9^ CFU/ animal [21], [27]. Reduced virulence of S19 in pregnant sows might suggest that the use of a heterologous strain might serve as a means to decrease the possibility of the LAV tropism of the placenta and fetal tissues, making it an attractive approach to minimize the unwanted side effects associated with the use of live attenuated vaccines.

The reproductive status and stage of gestation when a susceptible animal becomes infected (either by vaccination or infection) will have a different clinical manifestation. For example, if females become infected very early during pregnancy, sows will develop placentitis that can vary in their degree of magnitude and, if severe enough, can impair early fetal growth resulting in early embryonic death approximately 3–4 weeks post-infection [24]. In these cases, the only obvious clinical sign might be the return to estrus approximately 40 days after natural breeding due to the small amounts of uterine secretions that can be easily missed by the farmer [24]. If infection occurs later in the gestation period, placentitis with secondary impairment of oxygen and nutrients can result in stunted growth, fetal mortality, stillbirths or weak offspring. However, it is important to mention that spontaneous fetal loss is a common finding in pig farming throughout the world and this has to be differentiated from the potential adverse side effects of vaccination. It is estimated that following fertilization, 20–45% of normal pig conceptuses both at the preimplantation and at the mid to late gestation will not survive, and the exact cause for this is still unknown [28]. Therefore, a thorough evaluation consisting not only in determining the number of abortions, but also assessing the average numbers of piglets/sow, stillbirths, weak offspring, and fetal size was evaluated. In addition, histological evidence of a chronic inflammatory (i.e. placentitis or endometritis) process in reproductive tissues that cannot be detected during gross examination was confirmed by evaluating the uterus and placental tissues microscopically. During this study, no significant histologic evidence of adverse reproductive outcomes or the typical inflammatory changes with brucellosis were observed within vaccinated and control groups (Fig. 1). In this study, newborn deaths in all groups were evident and were the result of acute trauma, evidenced by the presence of hemorrhage during necropsy. It is well known that under normal conditions, newborn deaths increase when piglets are born from primiparous gilts as the ones used in this study, and is typically associated to post-farrowing stress and maternal infanticide ranging from fetal crushing to aggressive biting of the offspring [29], [30], [31].

The ability of the vaccine to colonize the placenta with subsequent vertical transmission to the fetus was also investigated. In swine, the transplacental transmission has been described in both naturally infected sows as well as in animals vaccinated with *Brucella suis* S2 [19], [32]. To assess, the potential of S19Δ*vjbR* or S19 vaccines to colonize the fetuses, multiple tissues were examined for the presence of residual vaccine strains. None of the 181 fetuses had any recoverable bacteria in either the lungs, stomach, gastric contents and umbilicus which are all preferential sites for isolating *Brucella* in fetuses [33], and this was further corroborated by unremarkable histology observed in the tissue sections from the piglets (Fig. 3), suggesting that S19 and S19 Δ*vjbR* in pregnant sows cannot cross the placental barrier when animals are vaccinated at mid-gestation.

Brucellosis in swine, like any other animal species, is characterized by the excretion of the bacteria into the environment through different secretions such as milk, urine or vaginal discharges [34], [35]. Shedding of vaccine strains through these routes is another important parameter to consider while developing a vaccine since contamination of the environment including water resources and pastures could potentially pose a risk to other non-target species residing in the same premises. In this study, the potential excretion of the vaccine strains in vaginal secretions was the focus, mainly because infected pregnant animals often excrete bacteria in their vaginal secretions [36]. None of the vaccinated animals shed any vaccine strains in vaginal secretions, suggesting that the possibility of cross-contamination to other animals residing in the same pastures might be limited. Interestingly, previous studies in swine infected with *B. abortus* Strain 544 demonstrated the presence of bacteria in vaginal swabs when animals were inoculated with a dose ranging from 5 × 10^8^ to 5 × 10^11^ CFU via the subcutaneous or intraconjunctival route [17] further indicating that the vaccine candidates are attenuated compared to *B. abortus* wild-type.

Despite the fact that undulant fever is one of the major symptoms of *Brucella* infection in humans [37], [38], the induction of fever associated with infection or vaccination has never been investigated in swine. In a recent study, an increase in body temperature was observed in pregnant heifers within 1 to 2 days following vaccination with *Brucella abortus* S19 [39]. To the best of our knowledge, this is the first study investigating body temperature fluctuations as a potential side effect of vaccination in swine. In this study, we monitored body temperature daily throughout the study (Fig. S1). None of the animals that received either S19 or the encapsulated and non-encapsulated S19 Δ*vjbR* developed fever. Whether fever can be used as a predictor of abortion or wild type infection in swine is yet to be studied, since none of the sows in this study aborted. However, vaccination did not induce fever in any of the gilts, further supporting the vaccine safety profile of both the encapsulated and non-encapsulated vaccines.

Although brucellosis is usually associated with reproductive failure, this is not pathognomonic nor the only clinical sign associated with the disease in swine. Brucellosis in swine has been considered to be somewhat different from the disease in ruminants with the development of a non-specific inflammatory process characterized by the formation of granulomas with extensive areas of necrosis that can be present in different organs such as lymph nodes, joints, bones and liver among others [36]. Experimental infection of *B. abortus* in swine has demonstrated that pigs are susceptible to infection, develop bacteremia and excrete bacteria into the environment up to 99 days post-inoculation [4], [17]. Following delivery, all sows were euthanized and multiple tissue sections were subjected to gross and microscopic evaluation to determine the presence of granulomatous lesions suggestive of potential subclinical brucellosis induced by the vaccine strains. None of the vaccinated animals regardless of the vaccine strain or formulation had any evidence of an ongoing inflammatory response that would indicate active infection or an adverse reaction to vaccination (Fig. 1). In addition, we also considered the possibility of the potential risk for human exposure while handling carcasses that could potentially harbor live organisms by enumerating the number of organisms present in different tissues following delivery. Seven weeks (46 to 52 days) post-vaccination, none of the vaccine strains were isolated from any of the major organs examined including lymph nodes, a typical site of localization of *Brucella* in swine, suggesting that handling of vaccinated carcasses after 7 weeks post-vaccination is safe. Based on the bacteriology, histopathology and Immune response data, vaccine clearance occurs between 6 and 7 weeks.

As the first step towards the characterization of the immune response of the vaccine candidates in sows, we investigated the humoral response associated with vaccination using RBT and iELISA. As a confirmatory test, iELISA is commonly employed following a positive RBT. During these studies, *Brucella* specific IgG and IgM responses peaked at two weeks post-vaccination and dropped significantly by six weeks except for the IgG response of encapsulated S19Δ*vjbR* group. These findings correlate with the unpublished results referenced in a study from the 40′s in which they indicate that swine vaccinated with S19 had a positive agglutination response that dropped rapidly by 4 weeks following vaccination [16]. Interestingly, the kinetics of a humoral response induced by S19 or S19Δ*vjbR* in swine is a transient, short-lived response compared to what it is typically observed in mice, cattle or red deer inoculated with S19 or S19Δ*vjbR* [8], [40], [41]. These results highlight a significant difference in the humoral response in swine compared to other animal species in which vaccination with S19 induce a long response that can last up to 38 weeks following vaccination [42]. Interestingly, the induction of such a short humoral response is not the case for swine infected with wild-type *B. suis*. Experimental infection of swine with 2 × 10^7^ CFU of *Brucella suis* biovar 2 through the conjunctival route results in a *Brucella*-specific IgG response that lasts approximately 21 weeks following inoculation [43]. Similarly, vaccination with S2 vaccine induces a humoral response that can persist for up to 21 weeks [19], [44]. Interestingly, inoculation of *B. abortus* wild-type in swine induces a shorter response compared to *B. suis* in swine that persists for 10 weeks following inoculation [17]. The identification of correlates of protective immunity for brucellosis vaccines still remains a subject of investigation and the determination of the immune response triggered by the different *Brucella* vaccines has been primarily studied in the mouse model and to a lesser degree in other species including cattle [45]. Mouse studies have revealed that the induction of cellular immunity is a key component for protection. However, the humoral response seems to also play an important role in protection, although the exact mechanism behind this observation has not been completely elucidated [46]. Whether this short humoral response is associated with lower levels of protective immunity or not, remains a subject of future investigation. Since S19 and S19Δ*vjbR* vaccination elicited a transient humoral response, this could potentially be used to our advantage to distinguish between vaccinated and naturally exposed animals. Naturally, infected swine will have a sustained response for at least 21 weeks (or 10 weeks if infected with *B. abortus*) as opposed to swine vaccinated with S19 or S19 Δ*vjbR* which will become negative after 6 weeks via RBT [43]*.* Practically, a screening test within 2 week and 4-week post-vaccination interval can be introduced in the field and serve as a DIVA (Differentiate Infected from Vaccinated Animals) strategy while vaccinating swine with either S19 or S19 Δ*vjbR.*

## 5 Conclusions

Overall, the vaccine candidates regardless of the formulation proved to be safe in pregnant swine and did not induce any undesirable side effect. Since no adverse pregnancy outcomes, tissue pathology or organism excretion was observed in these studies, efficacy studies in pregnant swine are the next logical step towards the development of a vaccine for swine. Future efficacy studies will also support or refute the use of alginate microencapsulation as a delivery system to improve vaccination efficacy.

## Declaration of Competing Interest

The authors declare the following financial interests/personal relationships which may be considered as potential competing interests: Allison Rice-Ficht, managing partner of NanoRelease Technologies (NRT), LLC Inc., has a 95% equity interest in NRT, a company involved in vaccine delivery platforms. The terms of this arrangement have been reviewed and approved by TXAgriLife Research and Texas A&M University in accordance with their conflict of interest policies.

## Figures and Tables

**Supplementary figure 1:**