# Supplementary material for: Vaccine safety studies of Brucella abortus S19 and S19ΔvjbR in pregnant swine
Source: Vaccine X. 2019 Aug 22;3:100041. doi: 10.1016/j.jvacx.2019.100041 (PMC6737346; doi:10.1016/j.jvacx.2019.100041)
Supplement: Supplementary data 1 [file mmc1.pdf]

Table S1. Gestion duration of vaccinated animals

Group	Animal number	Insemination date	Birth date	Gestation* duration	Gestation duration (Mean ± SD)[a]
S19	1	8/24/2014	12/13/2014	112	113.75 ± 1.5
	2	8/19/2014	12/11/2014	115	
	3	8/24/2014	12/14/2014	113	
	4	8/18/2014	12/10/2014	115	
S19 Δ*vjbR* encapsulated	1	8/19/2014	12/11/2014	115	114.5 ± 1.0
	2	8/22/2014	12/14/2014	115	
	3	8/18/2014	12/08/2014	113	
	4	8/18/2014	12/10/2014	115	
S19 Δ*vjbR* unencapsulated	1	8/18/2014	12/11/2014	116	114.75 ± 1.25
	2	8/19/2014	12/11/2014	115	
	3	8/19/2014	12/11/2014	115	
	4	8/19/2014	12/9/2014	113	
Control	1	8/22/2014	12/13/2014	114	114.0 ± 1.0
	2	9/7/2014	12/28/2014	113	
	3	8/22/2014	12/14/2014	115	

* Statistically no significant differences in gestation duration were observed between the groups. No significant differences were found using one-way ANOVA with Holm-Sidak's multiple comparison test (P>0.05).
