# Supplementary material for: Vaccine safety studies of Brucella abortus S19 and S19ΔvjbR in pregnant swine
Source: Vaccine X. 2019 Aug 22;3:100041. doi: 10.1016/j.jvacx.2019.100041 (PMC6737346; doi:10.1016/j.jvacx.2019.100041)
Supplement: Supplementary data 2 [file mmc2.pdf]

Table S2. Rose Bengal screening test (RBT) of samples collected from vaccinated gilts at 0, 2, 4- and 6-week post-vaccination.

Group	Animal Number	Pre-vaccination	2 week post vaccination	4 week post vaccination	6 Week post vaccination
S19	1	-	+++	++	-
	2	-	+++	n/a	-
	3	-	+++	+	-
	4	-	+++	++	-
S19 Δ*vjbR* encapsulated	1	-	+++	+	n/a
	2	-	++	++	-
	3	-	++	-	-
	4	-	++	n/a	-
S19 Δ*vjbR* unencapsultated	1	-	+++	+	-
	2	-	++++	+++	n/a
	3	-	++++	+++	-
	4	-	-	-	-
Control	1	-	-	-	-
	2	-	-	-	n/a
	3	-	-	-	-

n/a: collection of blood was not available

++++/+++ strong agglutination
++ mild agglutination
+ weak agglutination
- no agglutination
